# Isolated Double Orifice Mitral Valve in a Patient With Autosomal Dominant Polycystic Kidney Disease

**DOI:** 10.1016/j.case.2023.02.002

**Published:** 2023-04-04

**Authors:** Kyle Digrande, Jennifer Xu, Elizabeth H. Dineen, Andy Y. Lee

**Affiliations:** a Department of Medicine, School of Medicine, University of California, Irvine, Irvine, California; b Division of Cardiology, Department of Medicine, School of Medicine, University of California, Irvine, Irvine, California

**Keywords:** Congenital, Double orifice mitral valve

## Abstract

• DOMV can be diagnosed with TTE alone.
• DOMV can either be syndromic or isolated.
• Asymptomatic DOMV does not require treatment.
• The link between ADPKD and DOMV warrants more study.

## Graphical abstract

**Figure:**
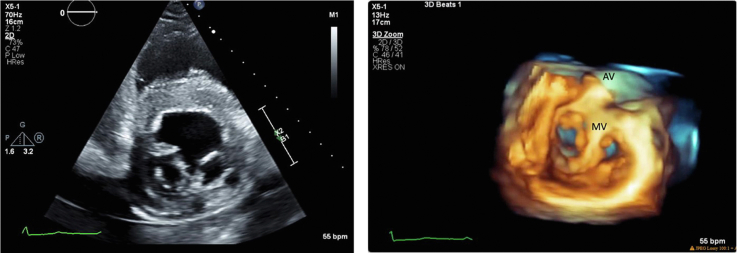

## Introduction

A double orifice mitral valve (DOMV), described as an accessory fibrous bridge between the anterior and posterior mitral valve leaflets that thereby creates 2 separate atrioventricular passages, is a rare congenital anomaly that has been associated with a number of inherited syndromes. Autosomal dominant polycystic kidney disease (ADPKD) is a congenital condition associated with chronic kidney disease as well as numerous extrarenal manifestations. While ADPKD has been linked to various congenital heart diseases including mitral valve prolapse and aortic regurgitation, there is no established association between ADPKD and DOMV. This is a reported case of an incidental DOMV in a patient with ADPKD during a kidney transplant evaluation.

## Case Presentation

**Figure 1:**
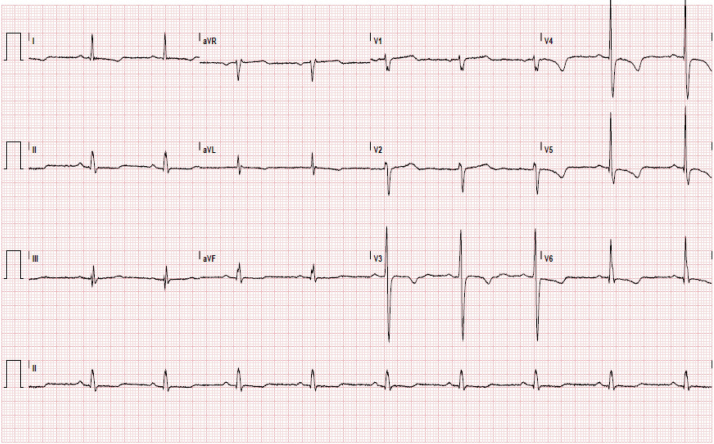
Twelve-lead electrocardiogram shows sinus bradycardia with T-wave flattening in the inferior leads as well as T-wave inversions in the anterolateral leads.

A 60-year-old male patient with end-stage renal disease due to ADPKD, previous hemorrhagic stroke secondary to a brain aneurysm, and hypertension presented to the cardiology clinic for cardiovascular risk stratification prior to kidney transplantation. The patient endorsed being able to walk at least 30 minutes daily without feeling limited by shortness of breath or fatigue. The patient denied any family history of cardiac abnormalities. The patient had a blood pressure of 147/92 mm Hg, a heart rate of 52 beats per minute, body mass index of 22.57 kg/m^2^, and 100% arterial O_2_ saturation by oximetry. Physical exam revealed a 1/6 precordial diastolic murmur. Electrocardiography demonstrated sinus bradycardia with T-wave flattening in the inferior leads as well as T-wave inversions in the anterolateral leads (Figure 1).

**Figure 2:**
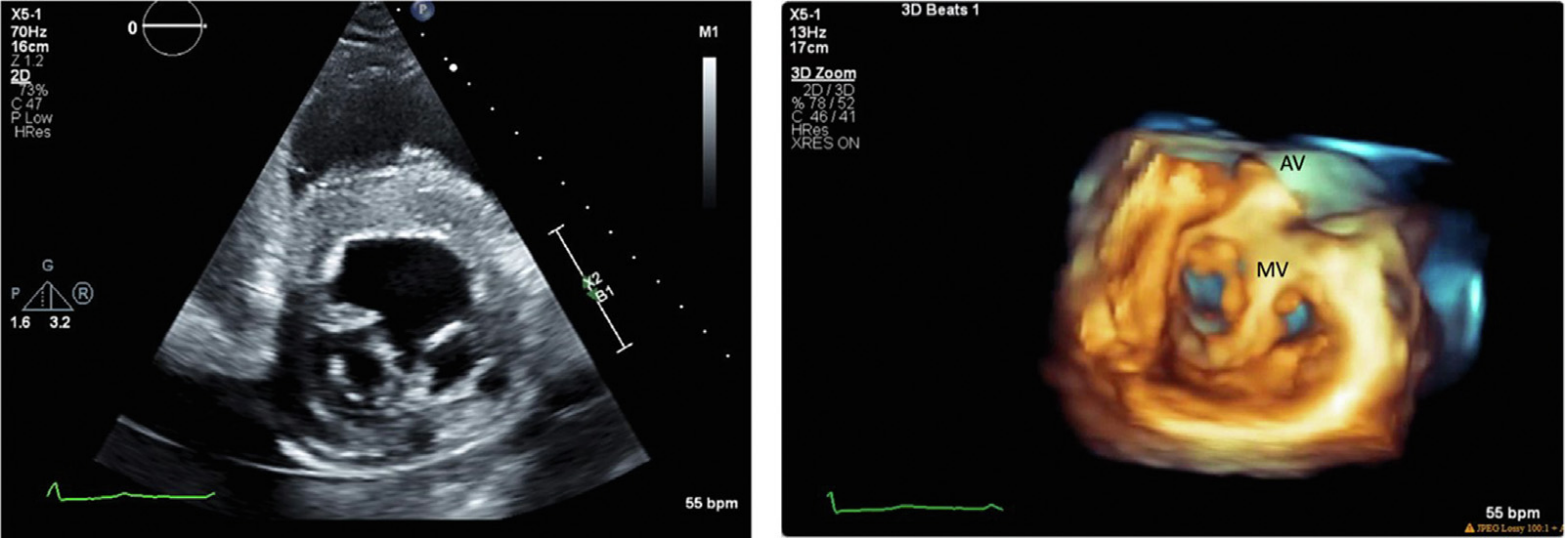
Two-dimensional TTE (*left*) and three-dimensional TTE (*right*), parasternal short-axis view at the level of the mitral annulus (diastolic phase), both showing the mitral valve at the annular level. There is a tissue bridge bisecting the center of the opening between the anterior and posterior leaflets into 2 similarly sized circular orifices. Findings are consistent with a complete bridge–type DOMV. *AV*, Aortic valve; *LV*, left ventricle; *MV*, mitral valve; *RV*, right ventricle.

**Figure 3:**
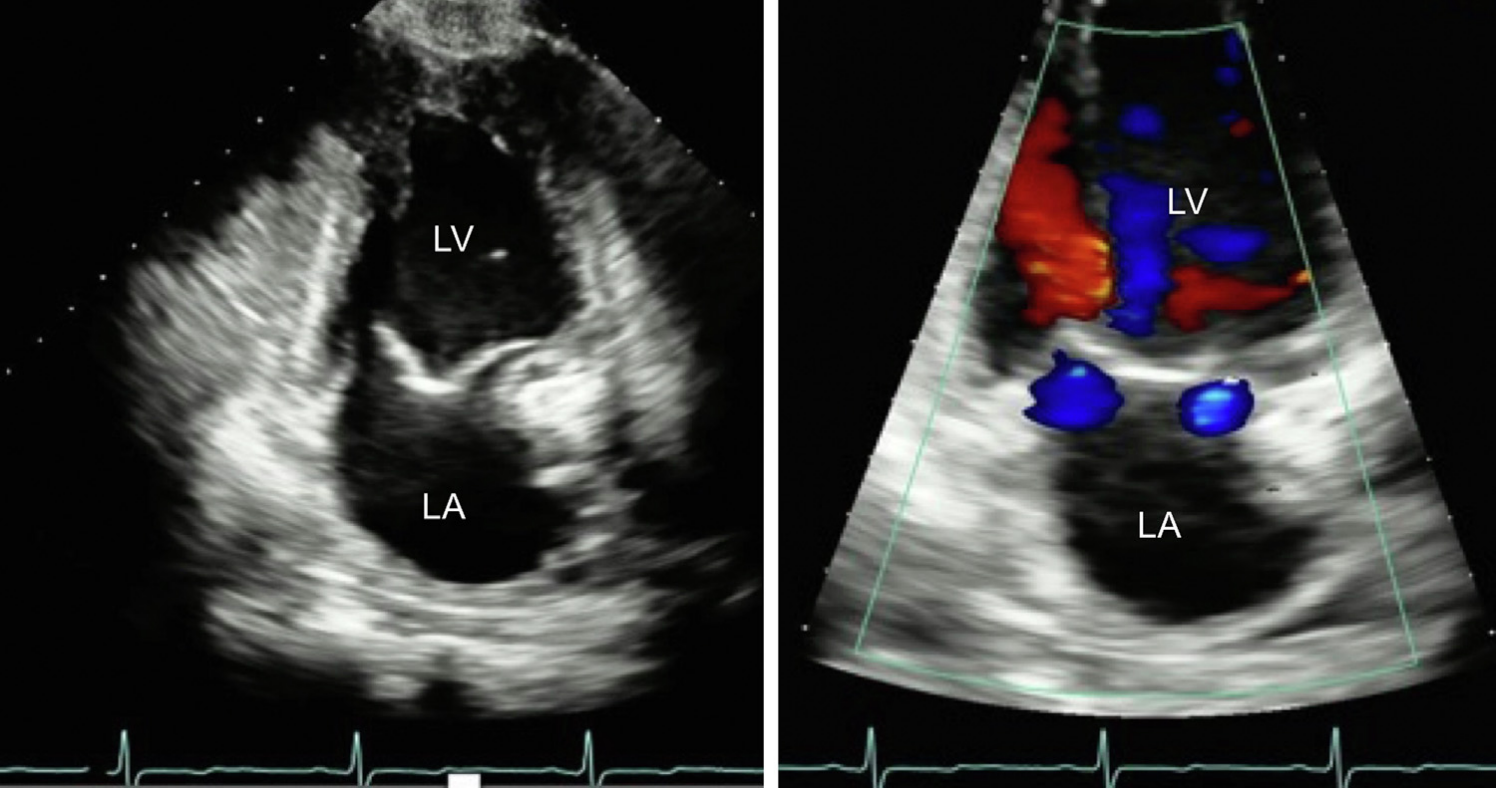
Two dimensional TTE, apical 2-chamber view, early systolic phase, without (*left*) and with (*right*) color flow Doppler demonstrates normal LV size and function with mild mitral regurgitation in both orifices. *LA*, Left atrium; *LV*, left ventricle.

**Figure 4:**
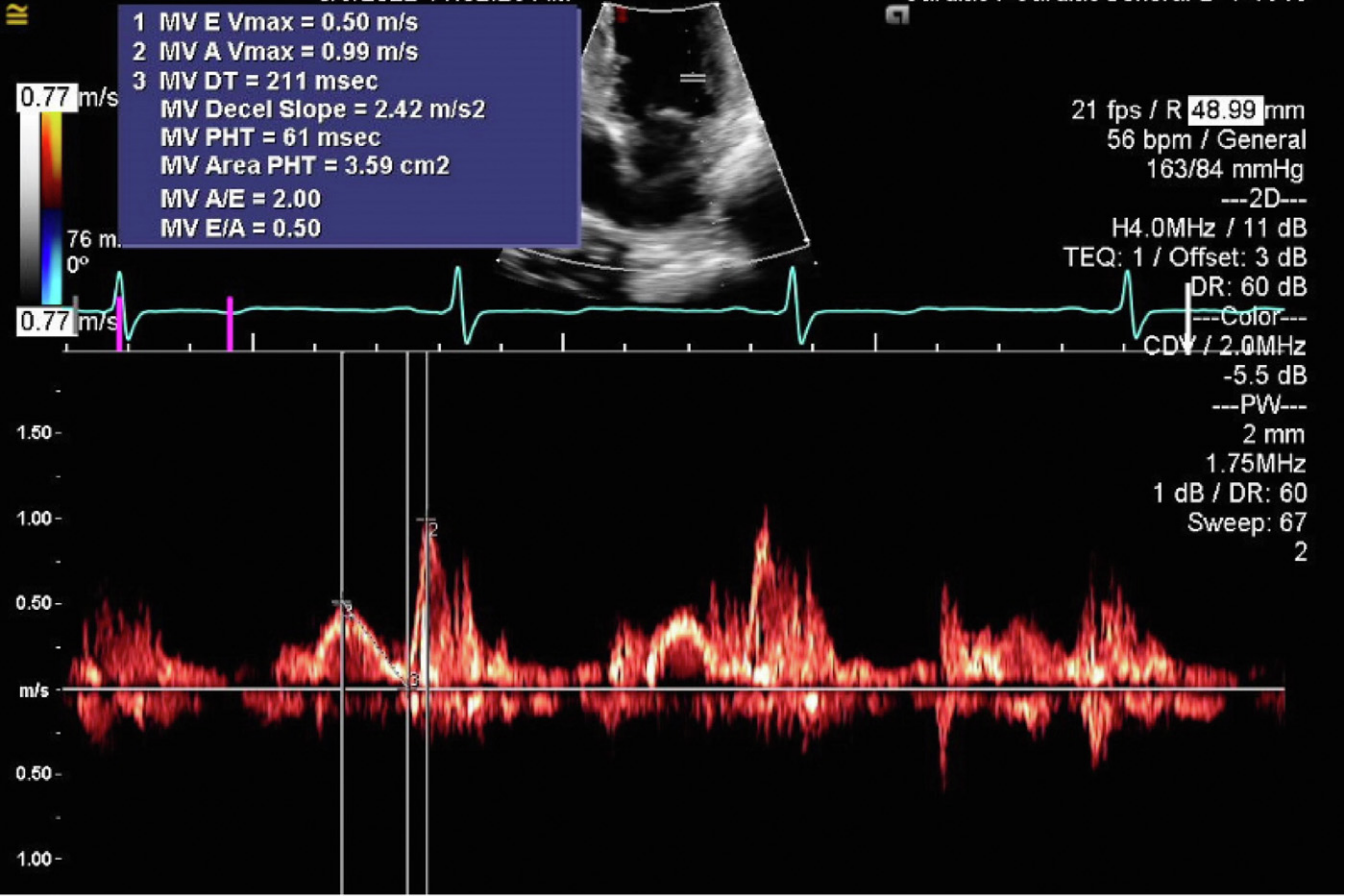
Two-dimensional TTE, apical 2-chamber view, pulsed-wave Doppler at the level of the mitral valve leaflet tip, demonstrating the mitral valve inflow pattern. There is A-wave dominance, suggestive of mild mitral regurgitation.

**Figure 5:**
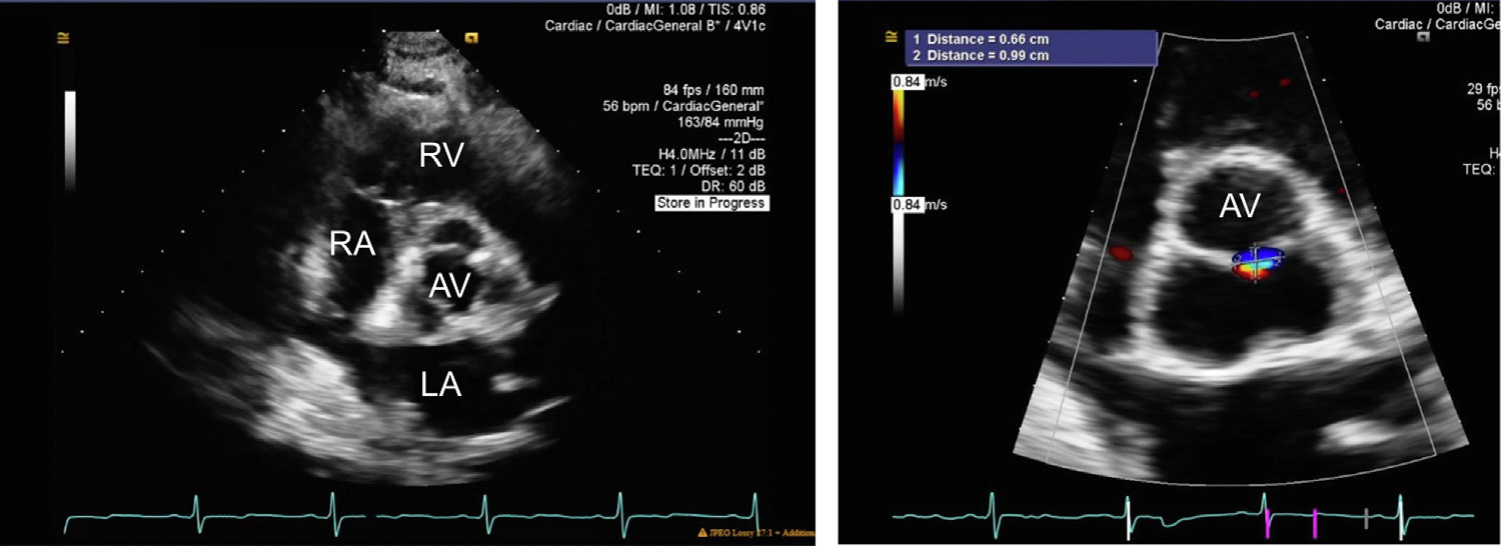
Two-dimensional TTE, parasternal short-axis view at the level of the aortic valve, diastolic phase, without (*left*) and zoomed with (*right*) color flow Doppler, demonstrates a tricuspid aortic valve with mild aortic regurgitation. *AV*, Aortic valve; *LA*, left atrium; *RA*, right atrium; *RV*, right ventricle.

**Figure 6:**
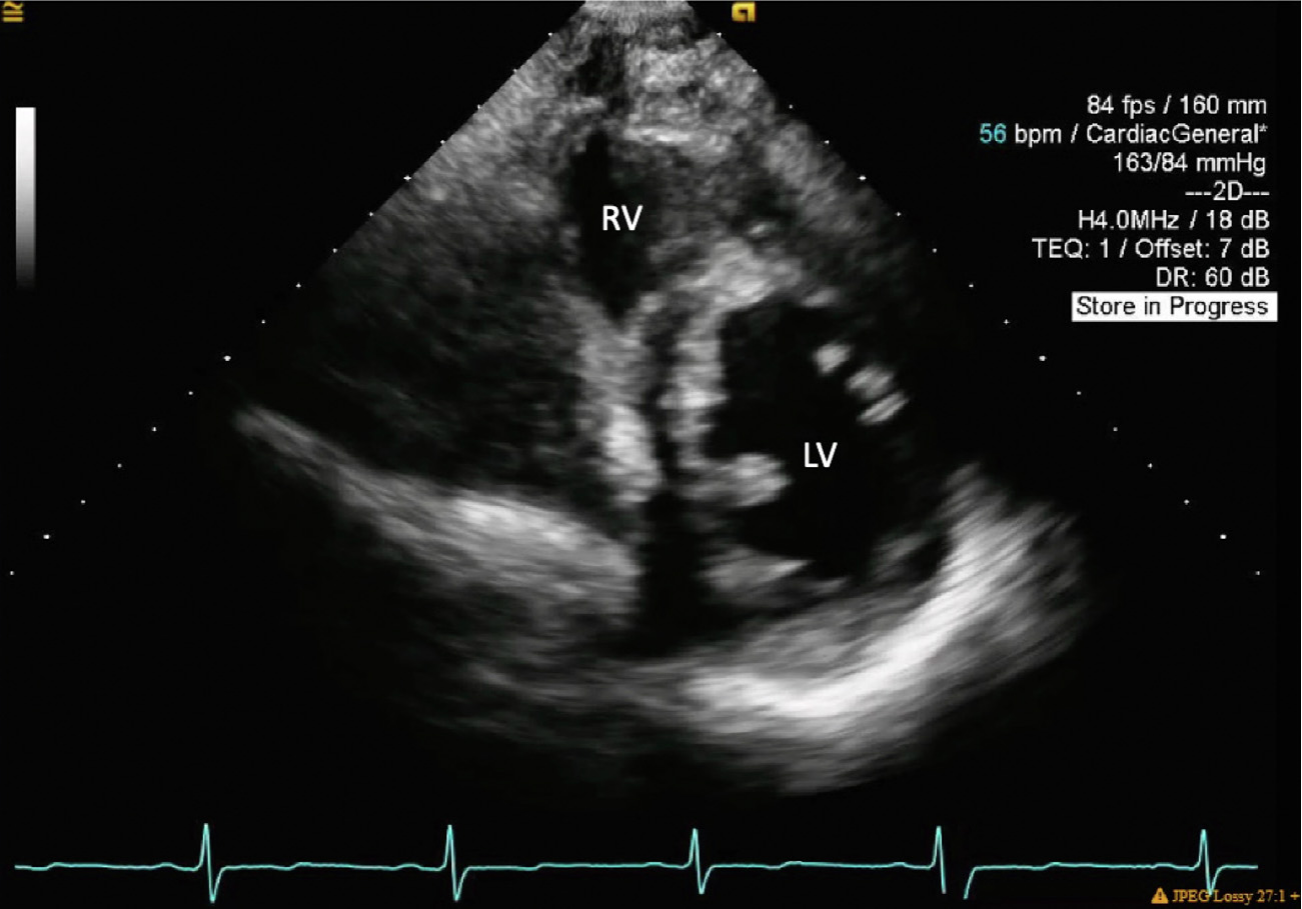
Two-dimensional TTE, parasternal short-axis view, midventricular, diastolic phase, demonstrates normal-appearing papillary muscles and conventional attachment of the papillary muscles to the chordae tendineae. *LV*, Left ventricle; *RV*, right ventricle.

A transthoracic echocardiogram (TTE) was obtained: the left ventricle internal dimension in diastole was 5.4 cm; interventricular septum thickness, 1.3 cm; left ventricular posterior wall, 1.4 cm; and left ventricular ejection fraction, 56% by the biplane method. All segments of the left ventricular wall were scored as normal. The parasternal short-axis view at the level of the mitral valve annulus demonstrated a tissue bridge bisecting the center of the opening between the anterior and posterior leaflets into 2 similarly sized, circular orifices (Figure 2, Videos 1 and 2). Mild mitral regurgitation (with mitral inflow A-wave dominance) was observed to a similar degree in both orifices (Figures 3 and 4, Video 3), and mild mitral stenosis was seen (mean inflow gradient 1 mm Hg at a heart rate of 55 beats per minute and mitral valve area of 3.6 cm^2^). These findings were consistent with a complete bridge–type DOMV. The echocardiogram was also remarkable for a dilated aortic root of 4.2 cm in diameter, a trileaflet aortic valve with mild aortic regurgitation (vena contracta width of 0.2 cm and jet area 1.4% of the left ventricular outflow tract cross-sectional area; Figure 5, Video 4). No additional defects of the mitral valve apparatus were identified, with normal-appearing papillary muscles and conventional attachment of the papillary muscles to the chordae tendineae (Figure 6, Video 5). Numerous echogenic cysts were visualized in the liver that were causing compression of the right atrium (Video 6). No other congenital abnormalities were identified. Myocardial perfusion imaging with a radioisotope was obtained as part of the pretransplant workup and demonstrated no reversible perfusion defects to suggest ischemia. The patient was seen in the cardiology clinic and advised to continue surveillance imaging of the DOMV as well as the aortic root dilation but otherwise was deemed an appropriate candidate from a cardiovascular standpoint to proceed with renal transplant.

## Discussion

Congenital DOMV is an exceedingly rare congenital anomaly, with a prior autopsy study demonstrating an incidence of 1% of cases of congenital heart disease.^1^ It is often an incidental finding without preceding symptoms, although late presentation in some cases may be associated with significant mitral stenosis or regurgitation and the complications associated with those valvular abnormalities including congestive heart failure and cardiac arrhythmias.^2^ Double orifice mitral valve often presents with associated congenital heart defects, which may include atrial or ventricular septal defects, coarctation of the aorta, bicuspid aortic valve, or patent ductus arteriosus^1^ and has been identified in Shone complex.^3^

Double orifice mitral valve may be difficult to detect on two-dimensional TTE without excellent image quality; this was demonstrated in this case as multiple previous echocardiograms did not demonstrate this finding. The defect is best visualized in the parasternal short-axis view, and care should be taken to evaluate for other left-sided obstructive cardiac lesions given the association with DOMV. Three-dimensional echocardiography has been shown to identify and characterize the abnormality and assess its anatomic impact on the mitral valve.^2^ Further imaging with transesophageal echocardiography or cardiac magnetic resonance imaging is not typically necessary.

Based on the echocardiographic appearance, the defect may be subdivided into 3 categories: (1) incomplete bridge type, when a small strand of fibrous tissue connects only the tips of the anterior and posterior leaflets, (2) complete bridge type, characterized by a fibrous bridge in the plane of the mitral valve leaflets, dividing the mitral valve opening into 2 parts that may be equal or unequal, or (3) hole type, defined as an accessory orifice surrounded by leaflet tissue that may have been a chordal ring.^4^ It is suspected that these subcategories represent variations of an anomaly in embryogenesis involving the left lateral, inferior, and superior endocardial cushions.^2^ The hole type of DOMV is likely from an abnormal fusion of the inferior and superior endocardial cushions, whereas the complete bridging type is presumably an abnormal adhesion of the lateral endocardial cushion and the left border of the superior and inferior cushions.^5^ Endocardial cushion defects are also associated with anomalies of the left ventricular-aortic junction, which may explain the link between DOMV and defects such as coarctation of the aorta or aortic stenosis.^5^

Management of DOMV is dictated by presence of symptoms. For asymptomatic patients, periodic surveillance echocardiography is recommended, although no formal guidelines exist. For patients with resultant symptomatic mitral regurgitation or stenosis, surgical valve replacement or even surgical repair by division of the bridging tissue may be indicated.^6^

While DOMV has been associated with genetic conditions such as Down syndrome and Turner syndrome,^1^ it has never been linked to ADPKD in the available literature. Autosomal dominant polycystic kidney disease is a genetic disorder characterized by numerous renal cysts, which eventually results in kidney failure.^7^ Most cases of ADPKD arise from pathogenic variants in either the PKD1 or PKD2 genes, which encode the proteins polycystin-1 and polycystin-2, respectively.^7^ Both proteins are present in the kidney tubular epithelial cells, cholangiocytes, vascular endothelial and smooth muscles, and cardiomyocytes.^7^ In cardiac tissue, the polycystin proteins have been implicated in left-right axis cardiac development as well as development of the interventricular and interatrial septa during embryogenesis.^7^ Clinically, congenital heart disease has been reported to be more prevalent in children with ADPKD compared with the normal population. These cardiac conditions include atrial septal defects, ventricular septal defects, patent ductus arteriosus, coarctation of the aorta, tetralogy of Fallot, and Ebstein anomaly.^7^ It has been suggested that the polycystin-1 protein is heavily expressed in the aortic outflow tract, atrial appendages, and endocardial cushions and heart valves.^7^ One may speculate that the involvement of polycystin-1 in cardiac development may potentially link the development of cardiac anomalies including DOMV to ADPKD. Regardless of this possibility, this case demonstrates the unique occurrence of 2 rare conditions that may warrant further investigation if similar cases are identified.

## Conclusion

Double orifice mitral valve is a rare cardiac anomaly with a unique echocardiographic appearance. If found incidentally without significant valvular dysfunction or hemodynamic effects related to the abnormalities, surveillance echocardiography is recommended. The association of ADPKD with DOMV is unknown.

## Ethics Statement

The authors declare that the work described has been carried out in accordance with The Code of Ethics of the World Medical Association (Declaration of Helsinki) for experiments involving humans.

## Consent Statement

Complete written informed consent was obtained from the patient (or appropriate parent, guardian, or power of attorney) for the publication of this study and accompanying images.

## Funding Statement

The authors declare that this report did not receive any specific grant from funding agencies in the public, commercial, or not-for-profit sectors.

## Disclosure Statement

The authors report no conflict of interest.

## Supplementary Data

Video 1 Two-dimensional TTE, parasternal short-axis view, showing the mitral valve at the annular level. There is a tissue bridge bisecting the center of the opening between the anterior and posterior leaflets into 2 similarly sized, circular orifices. Findings are consistent with a complete bridge–type DOMV.

Video 2 Three-dimensional TTE, parasternal short-axis view, showing the mitral valve at the level of the annulus. There is a tissue bridge bisecting the center of the opening between the anterior and posterior leaflets into 2 similarly sized, circular orifices. Findings are consistent with a complete bridge–type DOMV.

Video 3 Two-dimensional TTE, oblique apical view without (*left*) and with (*right*) color flow Doppler, demonstrates mild mitral regurgitation.

Video 4 Two-dimensional TTE, parasternal long-axis view, demonstrates a dilated sinus of Valsalva (4.2 cm), increased LV myocardial wall thickness, and short chordae tendineae or papillary muscle attached directly to the base of the mitral valve leaflet (this finding was not seen on other views).

Video 5 Two-dimensional TTE, parasternal short-axis view at the midventricular level, demonstrates normal-appearing papillary muscles and conventional attachment of the papillary muscles to the chordae tendineae. Image quality is reduced from respiratory motion artifact.

Video 6 Two-dimensional TTE, apical 4-chamber view (foreshortened apex) with high depth, demonstrates numerous echogenic cysts in the liver causing compression of the right atrium.
